# Obstetrics

**Published:** 1859-01

**Authors:** 


### OBSTETRICS.

### IV.—*On the Puerperal Diseases observed in the Charité of Berlin.* By PROF. VIRCHOW.

Puerperal diseases had prevailed in the Charité, without cessation, from the autumn of 1856, to the middle of March, 1858, during which time eighty-three fatal cases occurred. As in the epidemic of 1846-47, the frequency and severity of cases was greater in winter than in spring, a circumstance in which puerperal fever differs from hospital gangrene, which occurs particularly in spring. According to the author's opinion, it is possible that this aggravation is owing to a concentration of the miasmata in the wards, ventilation being only sparingly used for fear of producing colds.

In two cases only the very insignificant local changes were in no proportion to the severity of the symptoms; but it must be remarked, that these cases did not coincide with the maximum intensity of the epidemic, and that both patients had already, previous to their confinement, suffered with nervous symptoms. The rapidly fatal termination was thus not attributable to an intense alteration of the blood, but perhaps to a special predisposition of the nervous system. Acute endocarditis was observed in a large number of cases; it affected particularly the mitral valve, and produced in its tissue a peculiar softening and small crevices. Twice the fragments, detached from the valve by the current of blood, had produced obstructions in the capillary vessels and circumscribed foci of inflammation; in one of these cases small purulent collections were found in the muscular tissue of the heart, in the kidneys, the spleen, the liver, and both eyes; the uterus was normal. In the centre of these abscesses, as well as in a small artery, fragments of the mitral valve were found by chemical analysis. Similar anatomical changes were observed in four cases. From this circumstance, Prof. Virchow draws the conclusion, that the heart is frequently the point of issue of these so-called metastatic abscesses, and that, in a certain number of cases, they are the consequence of capillary embolia, and not of pyæmia. One of the patients succumbed suddenly to softening of the heart; it is, therefore, necessary to examine this organ attentively in every case where the state of the abdomen does not account satisfactorily for the gravity of the symptoms. In a patient who had suffered with hemiplegia of the left side, the veins of the arachnoid of the right hemisphere were obliterated by clots, and a great part of the cerebral substance was œdematous, and had ecchymotic spots disseminated through it. These lesions were, however, exceptional; in most cases peritonitis was present, and was often exclusively observed. It was found in two different forms; the first was superficial, and the exudation, either plastic or purulent, was deposited on the surface of the peritoneum. In the other much severer form the subserous areolar tissue was implicated, and transformed into a detritus mixed with pus. This form is identical with the diphtheritis of the internal surface of the uterus, which most frequently occurs on the surface of insertion of the placenta.

The uterus was inflamed only in one case; the inflammation was very extensive, and had terminated in gangrene. Inflammation of the ovaries was more frequent, and also assumed two forms analogous to those of the peritonitis. In one there existed a superficial hyperæmia, followed by abscesses in one or more follicles, and consequent effusion of pus into the peritoneum; in the other, a diffuse inflammation of the parenchyma was observed, producing considerable swelling and softening of the ovary.

Some stages of the epidemic were characterized by the frequency of phlebitis, nearly always accompanied by metastases, which Prof. Virchow ascribes to embolia. The angioleucitis was, on the contrary, rarely complicated. Prof. Virchow explains this difference by the arrest of the migrating particles in the lymphatic glands, where they become a source of inflammation.

The inflammation of the vessels is most frequently the consequence of ruptures of the perineum, of the vagina, and of the neck of the uterus, and is often complicated with extensive gangrene of the areolar tissue in the true pelvis and iliac fossæ. Prof. Virchow observed a case of this kind at Würzburg, in which the retro-peritoneal inflammation extended from the organs of generation upward to the diaphragm. (*Monatschrift für Geburtskunde*, xi. 409.)

